# Case 6 / 2015 - A 27-Year-Old Male Patient with Double Aortic and Pulmonary Valve Lesion after Double Valvotomy in Childhood

**DOI:** 10.5935/abc.20140214

**Published:** 2015-08

**Authors:** Edmar Atik

**Affiliations:** Clínica Privada Dr. Edmar Atik, São Paulo, SP - Brazil

**Keywords:** Pulmonary Valve Stenosis, Aortic Valve Stenosis, Balloon Valvoplasty, Heart Defects, Congenital

**Clinical data**: The patient underwent correction of congenital pulmonary and aortic valve stenosis, both with manifestations, at 7 years of age. Afterwards, there was progressive residual lesion of both valves, with predominance of regurgitation, and development of acute arrhythmias such as paroxysmal atrial fibrillation, in addition to ventricular extrasystoles. The obstructions were significant, with pressure gradients of 90 and 60 mmHg in the pulmonary and aortic valves, respectively. The patient was asymptomatic, and the loud systolic murmur was accompanied by thrill all over the precordium and neck vessels. There was right ventricular overload on the electrocardiogram (ECG). Double valvoplasty in the three-leaflet valves resulted in a good anatomical solution initially. To date, the patient reports shortness of breath on moderate exertion and precordial palpitations. He is on antiarrhythmic drug (amiodarone), after atrial fibrillation was controlled.

**Physical examination**: Good general state of health, normal breathing, acyanotic, normal pulses. Weight: 77 kg; height: 181 cm; blood pressure (BP): 110/70 mmHg; and heart rate (HR): 51 bpm. The aorta (Ao) was moderately palpable on the suprasternal notch.

The apical impulse was not palpable on the precordium, and there were mild systolic impulses on the left sternal border. Heart sounds were normal, and there was a grade 1-2/4 coarse systolic murmur in the pulmonic and aortic areas, and a grade 1-2/4 coarse diastolic murmur along the left sternal border. The liver was not palpable and the lungs were clear to auscultation.

## Laboratory tests:

**Figure 1:**
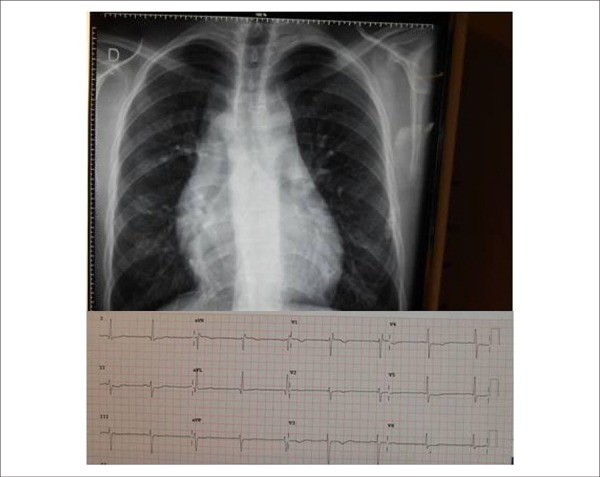
Chest radiograph shows moderate enlargement of the cardiac silhouette. Electrocardiogram shows left anterior hemiblock, with no signs of cardiac overload.

**Electrocardiogram** showed normal sinus rhythm and signs of left anterior hemiblock, with no chamber overload, and normal ventricular repolarization. PA: +20º, QRSA: -60º, TA: +40º. QRS complex duration was 0.11", PR = 0.16" and QTc = 0.45" (Figure 1).

**Chest radiograph** showed moderately enlarged cardiac silhouette due to enlarged atrial and ventricular arches; the pulmonary vascular network was normal. Cardiomegaly progressed since the surgical correction, and the current cardiothoracic ratio was 0.58 (Figure 1).

**Echocardiogram **showed dilated right and left cardiac chambers (right ventricle − RV = 35, left atrium − LA = 46; left ventricle − LV = 64; Ao = 31 mm); RV ejection fraction (RVEF) of 53% (Simpson’s method); LV ejection fraction (LVEF) of 58%; RV- pulmonary trunk (PT) pressure gradient of 14 mmHg; LV - Ao pressure gradient of 15 mmHg; and severe pulmonary and aortic regurgitation. The pulmonary systolic pressure was 40 mmHg. Ascending aorta and PT dilatation (40-mm diameter).

**Figure 2:**
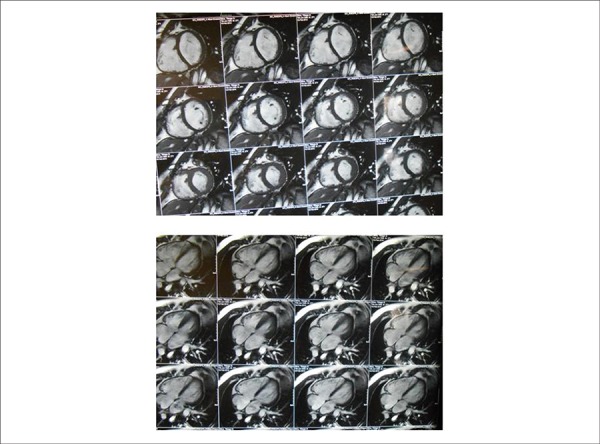
Magnetic resonance imaging shows clear enlargement of the right and left ventricles, with preserved function of both, in four-chamber and cross-sectional views.

**Magnetic resonance imaging** (Figure 2) also showed enlargement of right and left cardiac chambers with preserved ventricular function. RV end-diastolic volume of 200 mL/m^2,^ LV end-diastolic volume of 211 mL/m^2^, RVEF of 54% and LVEF of 58%. Ascending aorta of 45 mm, and PT of 36 mm.

**24-hour dynamic ECG (Holter monitoring)** showed 627 ventricular extrasystoles and 121 supraventricular extrasystoles. Two episodes of non-sustained ventricular tachycardia with 6 complexes and two of atrial tachycardia with 16 complexes.

**Clinical diagnosis:** Double pulmonary and aortic valve lesion with manifestations, showing signs of progressive dilatation of both ventricles, in late course after surgical correction in childhood.

**Clinical reasoning: **during the course of the disease, the clinical elements were consistent with the diagnosis of double pulmonary and aortic valve lesion, with predominance of the first. Shortness of breath and development of ventricular arrhythmias and paroxysmal atrial fibrillation are related to the residual lesions, which progressively increased since childhood. Noteworthy, despite the evident biventricular dilatation, no electrical overload of these chambers was observed. Perhaps both ventricular dilatations had electrically counterbalanced one another.

**Differential diagnosis:** Concomitant lesion of both semilunar valves as congenital defects is usually associated with some genetic syndrome, which was not the case. Residual lesions of both valves commonly occur after correction of obstructive defects alone or in association, as occurs in the tetralogy of Fallot.

**Management:** in view of the progression of the residual defects with excessive dilatation of both ventricles, although with biventricular function still preserved, a surgical approach was chosen with valve replacement for a mechanical prosthetic valve in the aortic position and for a biological prosthetic valve in the pulmonary position. Because of the ascending aorta dilatation, a dacron tube was inserted inside the vessel.

**Commentaries: **residual valve regurgitation after surgical or percutaneous valvotomy, whether pulmonary or aortic, has become a common outcome that requires the performance of other operative techniques, such as Ross’ technique. It is estimated that approximately 30% of these patients undergoing heart valve correction require surgical reintervention to prevent further progression of the heart valve defects, which ultimately result in ventricular dilatation and dysfunction.

The reintervention usually implies the need for valve replacement. The values currently recommended to prevent further deterioration of the ventricular function are 120 mL/m^2^ for the end-diastolic volume and of 90 mL/m^2^ for the end-systolic volume. In practice, however, we have observed much higher values until surgical reintervention is indicated, as incidentally occurred in the present case. Ideally, these patients should be duly monitored, in order to follow the parameters recommended for a more favorable outcome in the long term.

